# *catena*-Poly[[(*N*,*N*-di­methyl­cyanamide-κ*N*)lithium]-μ_3_-bromido]

**DOI:** 10.1107/S1600536814001652

**Published:** 2014-01-29

**Authors:** Qianwen Xie, Hongbo Tong, Meisu Zhou

**Affiliations:** a Institute of Applied Chemistry, Shanxi University, Taiyuan 030006, People’s Republic of China

## Abstract

The title complex, [LiBr(C_3_H_6_N_2_)]_*n*_, is the unexpected product of a reaction beteween (Dipp)N(Li)SiMe_3_ (Dipp = 2,6-diiso­propyl­phen­yl), Me_2_NCN and CuBr. The compound is a one-dimensional polymer with a step structure derived from the association of inversion dimers, formed by bromido ligands bridging two Li^+^ cations, each of which carries a di­methyl­cyanamide ligand. The planar (LiBr)_2_ unit of the polymer core has a regular rhombic shape [Li—Br—Li 77.55 (16)° and Br—Li—Br 102.45 (16)°]. These (LiBr·NCNMe_2_)_2_ dimers represent the repeat unit of a polymer system propagated by additional Br—Li and Li—Br bonds generating an infinite step structure along the *a-*axis direction.

## Related literature   

For examples of lithium halides solvated by Lewis bases, see: Snaith & Wright (1995 ▶); Mulvey (1991 ▶); Raston, Skelton *et al.* (1988 ▶), Raston, Whitaker & White (1988 ▶, 1989*a* ▶,*b* ▶); Edwards *et al.* (1993 ▶); Neumann *et al.* (1995 ▶); Gregory *et al.* (1991 ▶). For related crystal structures, see: Edwards *et al.* (1993 ▶); Raston, Skelton *et al.* (1988 ▶). A 1,3,5,7-tetra­aza­hepta­trien­yl–lithium salt was reported by Boesveld *et al.* (2009 ▶)
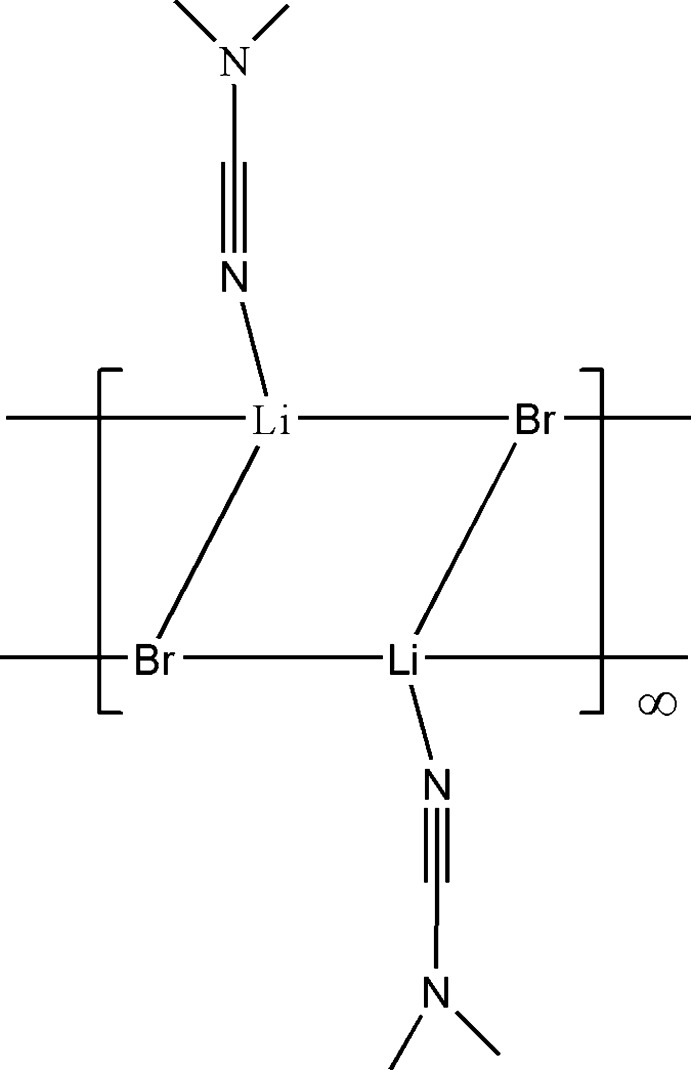


## Experimental   

### 

#### Crystal data  

[LiBr(C_3_H_6_N_2_)]
*M* *_r_* = 156.85
Monoclinic, 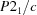

*a* = 4.2680 (8) Å
*b* = 17.214 (3) Å
*c* = 8.9685 (17) Å
β = 100.089 (3)°
*V* = 648.7 (2) Å^3^
*Z* = 4
Mo *K*α radiation
μ = 6.22 mm^−1^
*T* = 200 K
0.35 × 0.33 × 0.32 mm

#### Data collection  

Bruker SMART APEX CCD diffractometer
Absorption correction: multi-scan (*SADABS*; Bruker, 2000 ▶) *T*_min_ = 0.220, *T*_max_ = 0.241
3498 measured reflections
1140 independent reflections
965 reflections with *I* > 2σ(*I*)
*R*_int_ = 0.032

#### Refinement  

*R*[*F* ^2^ > 2σ(*F* ^2^)] = 0.025
*wR*(*F* ^2^) = 0.061
*S* = 1.03
1140 reflections
67 parameters
H-atom parameters constrained
Δρ_max_ = 0.57 e Å^−3^
Δρ_min_ = −0.38 e Å^−3^

### 

Data collection: *SMART* (Bruker, 2000 ▶); cell refinement: *SAINT* (Bruker, 2000 ▶); data reduction: *SAINT*; program(s) used to solve structure: *SHELXS97* (Sheldrick, 2008 ▶); program(s) used to refine structure: *SHELXL97* (Sheldrick, 2008 ▶); molecular graphics: *SHELXTL/PC* (Sheldrick, 2008 ▶); software used to prepare material for publication: *SHELXL97*.

## Supplementary Material

Crystal structure: contains datablock(s) I, global. DOI: 10.1107/S1600536814001652/sj5383sup1.cif

Structure factors: contains datablock(s) I. DOI: 10.1107/S1600536814001652/sj5383Isup2.hkl

CCDC reference:

Additional supporting information:  crystallographic information; 3D view; checkCIF report

## Figures and Tables

**Fig. 1.**
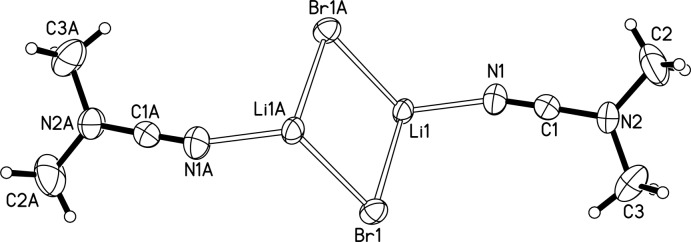
The basic repeat unit of the polymer (I) showing the atom numbering scheme with displacement ellipsoids drawn at the 50% probability level. Atoms labelled with a trailing A are related to the other atoms by the symmetry operation 1-x, 1-y, -z

**Fig. 2.**
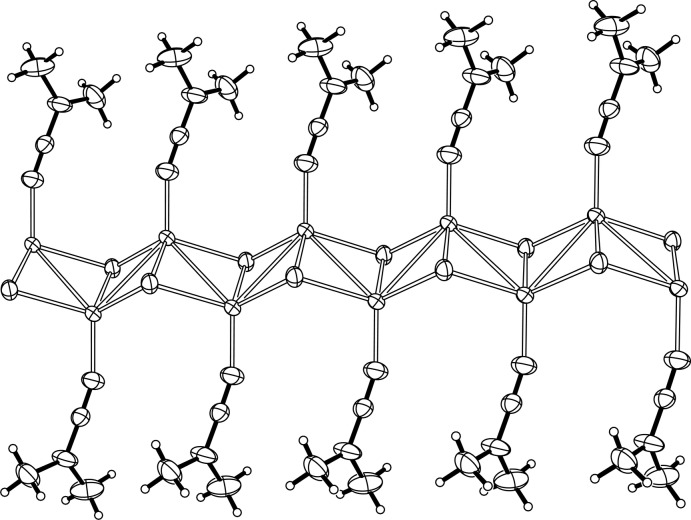
The expanded step polymeric structure of (I) viewed along the crystallographic c axis.

## supplementary crystallographic information

### 1.1. Synthesis and crystallization

Me_2_NCN (0.76 mL, 9.38 mmol) was added to a solution of (Dipp)N(Li)SiMe_3_ (0.60 g, 2.35 mmol) in Et_2_O (30 mL) at -78°C. The resulting mixture was warmed to *ca.* 25°C and stirred for overnight. The resulting mixture was added dropwise into a suspension of CuBr (0.34 g, 2.35 mmol) in Et_2_O (10 mL) at -78°C. The resulting mixture was warmed to *ca.* 25°C and stirred for 24 h, then filtered. The filtrate was concentrated in vacuo and stored at 20°C for ten days, yielding colorless crystals of the title compound (0.503 g, 68%) .

Anal. calcd. for C_6_H_12_Br_2_Li_2_N_4_(%): C, 22.96; H, 3.85; N, 17.85. Found: C, 22.93; H, 3.89; N, 17.87. All manipulations were performed under argon using standard Schlenk and vacuum line techniques. Et_2_O was dried and distilled over Na under argon prior to use. Elemental analysis is completely in agreement with the structure of the compound.

### 1.2. Refinement

Crystal data, data collection and structure refinement details are summarized in Table 1. The methyl H atoms were constrained to an ideal geometry, with C—H distances of 0.98 Å and *U*_iso_(H) = 1.5*U*_eq_(C).

### 2. Comment

Lithium halidies solvated by Lewis bases have been studied extensively in the past and the various crystal structures exhibit remarkable structural diversity (Snaith *et al.*, 1995; Mulvey, 1991, Gregory *et al.*, 1991). Monomers, dimers, tetra­mers, larger oligomers and polymers are known (Raston, Whitaker & White 1988, 1989*a*,*b*; Raston, Skelton *et al.* 1988; Edwards *et al.*,1993). Pyridines, chelating amines and Lewis bases containing oxygen usually serve as ligands (Neumann *et al.*, 1995). A 1,3,5,7-tetra­aza­heptatrienyl-lithium salt was reported by W. Marco Boesveld (Boesveld *et al.*, 2009) and we were attempting to synthesize a 1,3,5,7-tetra­aza­heptatrienylcopper complex by the reaction of (Dipp)N(Li)SiMe_3_ (Dipp = 2,6-diiso­propyl­phenyl), Me_2_NCN and CuBr. No copper complex was obtained but instead the title polymeric lithium complex (I), (C_6_H_12_Br_2_Li_2_N_4_)_∞_, was isolated from the reaction mixture. Here we present the synthesis and crystal structure of the complex (I).

A low-temperature X-ray crystallographic study shows the basic unit (Fig. 1) of the step structure of complex (I) is centrosymmetric, and to have a polymeric structure (Fig. 2) in the solid state. In the unit, atoms Li1, Br1, Li1A and Br1A are exactly co-planar and constitute a regular rhombic shape [Li—Br—Li 77.55 (16)° and Br—Li—Br 102.45 (16)° ]. The Li1—Br1 and Li1—N1 bond lengths are 2.543 (5) (*av*.) and 1.999 (5) Å. The bond angles N1—Li1—Br1, N1—Li1—Br1A are 113.1 (2) and 119.6 (2)°, respectively.

### Crystal data

**Table:**

[LiBr(C_3_H_6_N_2_)]	*F*(000) = 304
*M**_r_* = 156.85	*D*_x_ = 1.607 Mg m^−^^3^
Monoclinic, *P*2_1_/*c*	Mo *K*α radiation, λ = 0.71073 Å
*a* = 4.2680 (8) Å	Cell parameters from 1538 reflections
*b* = 17.214 (3) Å	θ = 2.4–26.5°
*c* = 8.9685 (17) Å	µ = 6.22 mm^−^^1^
β = 100.089 (3)°	*T* = 200 K
*V* = 648.7 (2) Å^3^	Block, colourless
*Z* = 4	0.35 × 0.33 × 0.32 mm

### Data collection

**Table:**

Bruker SMART APEX CCD diffractometer	1140 independent reflections
Radiation source: fine-focus sealed tube	965 reflections with *I* > 2σ(*I*)
Graphite monochromator	*R*_int_ = 0.032
ω scans	θ_max_ = 25.1°, θ_min_ = 2.4°
Absorption correction: multi-scan (*SADABS*; Bruker, 2000)	*h* = −5→3
*T*_min_ = 0.220, *T*_max_ = 0.241	*k* = −20→18
3498 measured reflections	*l* = −10→10

### Refinement

**Table:**

Refinement on *F*^2^	Secondary atom site location: difference Fourier map
Least-squares matrix: full	Hydrogen site location: inferred from neighbouring sites
*R*[*F*^2^ > 2σ(*F*^2^)] = 0.025	H-atom parameters constrained
*wR*(*F*^2^) = 0.061	*w* = 1/[σ^2^(*F*_o_^2^) + (0.0285*P*)^2^ + 0.3312*P*] where *P* = (*F*_o_^2^ + 2*F*_c_^2^)/3
*S* = 1.03	(Δ/σ)_max_ = 0.001
1140 reflections	Δρ_max_ = 0.57 e Å^−^^3^
67 parameters	Δρ_min_ = −0.38 e Å^−^^3^
0 restraints	Extinction correction: *SHELXL97* (Sheldrick, 2008), Fc^*^=kFc[1+0.001xFc^2^λ^3^/sin(2θ)]^-1/4^
Primary atom site location: structure-invariant direct methods	Extinction coefficient: 0.0053 (16)

### Special details

**Table:**

Geometry. All e.s.d.'s (except the e.s.d. in the dihedral angle between two l.s. planes) are estimated using the full covariance matrix. The cell e.s.d.'s are taken into account individually in the estimation of e.s.d.'s in distances, angles and torsion angles; correlations between e.s.d.'s in cell parameters are only used when they are defined by crystal symmetry. An approximate (isotropic) treatment of cell e.s.d.'s is used for estimating e.s.d.'s involving l.s. planes.
Refinement. Refinement of *F*^2^ against ALL reflections. The weighted *R*-factor *wR* and goodness of fit *S* are based on *F*^2^, conventional *R*-factors *R* are based on *F*, with *F* set to zero for negative *F*^2^. The threshold expression of *F*^2^ > σ(*F*^2^) is used only for calculating *R*-factors(gt) *etc*. and is not relevant to the choice of reflections for refinement. *R*-factors based on *F*^2^ are statistically about twice as large as those based on *F*, and *R*- factors based on ALL data will be even larger.

### Fractional atomic coordinates and isotropic or equivalent isotropic displacement parameters (Å^2^)

**Table:**

	*x*	*y*	*z*	*U*_iso_*/*U*_eq_	
Li1	0.2655 (11)	0.4372 (3)	0.0594 (6)	0.0289 (11)	
Br1	0.81425 (7)	0.485193 (18)	0.18525 (3)	0.03230 (16)	
N1	0.2914 (7)	0.32132 (16)	0.0517 (3)	0.0475 (8)	
N2	0.5102 (8)	0.18972 (16)	0.0853 (3)	0.0509 (8)	
C1	0.3896 (8)	0.25987 (19)	0.0667 (4)	0.0352 (8)	
C2	0.3986 (11)	0.1281 (2)	−0.0201 (5)	0.0672 (12)	
H2A	0.2384	0.1487	−0.1023	0.101*	
H2B	0.3037	0.0868	0.0327	0.101*	
H2C	0.5779	0.1070	−0.0621	0.101*	
C3	0.7254 (9)	0.1720 (2)	0.2260 (5)	0.0581 (11)	
H3A	0.7997	0.2206	0.2775	0.087*	
H3B	0.9081	0.1426	0.2035	0.087*	
H3C	0.6130	0.1410	0.2916	0.087*	

### Atomic displacement parameters (Å^2^)

**Table:**

	*U*^11^	*U*^22^	*U*^33^	*U*^12^	*U*^13^	*U*^23^
Li1	0.025 (3)	0.022 (3)	0.038 (3)	0.0034 (19)	0.001 (2)	0.004 (2)
Br1	0.0248 (2)	0.0371 (2)	0.0339 (2)	0.00073 (13)	0.00223 (13)	0.00497 (14)
N1	0.052 (2)	0.0287 (17)	0.057 (2)	0.0056 (14)	−0.0034 (15)	0.0025 (14)
N2	0.068 (2)	0.0252 (16)	0.053 (2)	0.0129 (14)	−0.0071 (16)	−0.0022 (14)
C1	0.035 (2)	0.032 (2)	0.0363 (19)	−0.0027 (15)	−0.0003 (14)	−0.0015 (14)
C2	0.108 (4)	0.036 (2)	0.062 (3)	−0.003 (2)	0.027 (2)	−0.0161 (19)
C3	0.050 (2)	0.058 (3)	0.065 (3)	0.0178 (19)	0.003 (2)	0.019 (2)

### Geometric parameters (Å, º)

**Table:**

Li1—N1	1.999 (5)	N2—C1	1.312 (4)
Li1—Br1^i^	2.535 (5)	N2—C2	1.445 (5)
Li1—Br1^ii^	2.541 (5)	N2—C3	1.457 (5)
Li1—Br1	2.553 (5)	C2—H2A	0.9800
Li1—Li1^iii^	3.179 (9)	C2—H2B	0.9800
Li1—Li1^ii^	3.251 (10)	C2—H2C	0.9800
Br1—Li1^iv^	2.535 (5)	C3—H3A	0.9800
Br1—Li1^ii^	2.541 (5)	C3—H3B	0.9800
N1—C1	1.137 (4)	C3—H3C	0.9800
			
N1—Li1—Br1^i^	113.1 (2)	C1—N1—Li1	161.0 (3)
N1—Li1—Br1^ii^	119.6 (2)	C1—N2—C2	121.0 (3)
Br1^i^—Li1—Br1^ii^	102.45 (16)	C1—N2—C3	118.4 (3)
N1—Li1—Br1	106.6 (2)	C2—N2—C3	119.9 (3)
Br1^i^—Li1—Br1	114.03 (19)	N1—C1—N2	178.5 (4)
Br1^ii^—Li1—Br1	100.67 (17)	N2—C2—H2A	109.5
N1—Li1—Li1^iii^	135.1 (3)	N2—C2—H2B	109.5
Br1^i^—Li1—Li1^iii^	51.30 (14)	H2A—C2—H2B	109.5
Br1^ii^—Li1—Li1^iii^	51.15 (14)	N2—C2—H2C	109.5
Br1—Li1—Li1^iii^	118.2 (2)	H2A—C2—H2C	109.5
N1—Li1—Li1^ii^	127.7 (3)	H2B—C2—H2C	109.5
Br1^i^—Li1—Li1^ii^	119.2 (2)	N2—C3—H3A	109.5
Br1^ii^—Li1—Li1^ii^	50.50 (13)	N2—C3—H3B	109.5
Br1—Li1—Li1^ii^	50.17 (13)	H3A—C3—H3B	109.5
Li1^iii^—Li1—Li1^ii^	83.2 (2)	N2—C3—H3C	109.5
Li1^iv^—Br1—Li1^ii^	77.55 (16)	H3A—C3—H3C	109.5
Li1^iv^—Br1—Li1	114.03 (19)	H3B—C3—H3C	109.5
Li1^ii^—Br1—Li1	79.33 (17)		

Symmetry codes: (i) *x*−1, *y*, *z*; (ii) −*x*+1, −*y*+1, −*z*; (iii) −*x*, −*y*+1, −*z*; (iv) *x*+1, *y*, *z*.
